# IMPROVING CARE THROUGH IMPROV: RESULTS OF A PILOT TEST WITH FAMILY AND FRIENDS OF PERSONS LIVING WITH DEMENTIA

**DOI:** 10.1093/geroni/igae098.4302

**Published:** 2024-12-31

**Authors:** Candace Kemp, Jennifer Morgan, Emerald Anglin, Celeste Greene, Crystal Williams, Amanda Williams

**Affiliations:** Georgia State University, Atlanta, Georgia, United States; Georgia State University, Atlanta, Georgia, United States; Georgia State University, Atlanta, Georgia, United States; Gerontology Institute at Georgia State University, Atlanta, Georgia, United States; Georgia State University, Atlanta, Georgia, United States; Dad’s Garage Theatre Company, Decatur, Georgia, United States

## Abstract

Evidence suggests that the use of improvisational (improv) theatre techniques in the dementia care context can have positive outcomes for care partners, persons living with dementia, and care relationships. We refined and pilot tested the feasibility, acceptability, and preliminary efficacy of the training program, “Improving Care through Improv,” which incorporates improv fundamentals with practical dementia care knowledge, among family and friends of persons living with moderate dementia. We used multiple methods to assess the program, including a no-control pre-post design trial involving surveys and focus groups. Participants received in-person training co-designed and co-led by an improv educator and a gerontologist during weekly 2-hour group sessions held over a 4-week period. We assessed the primary outcome (caregiver mastery) and exploratory outcomes (perceived stress, anxiety, caregiver burden, perceived ability to manage behaviors, and care recipient’s quality of life) at baseline; upon program completion, and 3 months post baseline. Forty care partners participated in the intervention, completed all three surveys, and participated in focus groups. Recruitment, enrollment, and completion rates alongside quantitative and qualitative findings indicate high levels of feasibility and acceptability. Dependent sample t-tests between times 1 and 3 showed significant improvement in caregiver mastery and significant reductions in perceived stress and depression. Participants characterized the program as “needed” and “very helpful.” Three months post baseline, 100% reported using the skills taught, with most saying that they used them “frequently” or “nearly always.” Findings demonstrate improv’s promise for improving dementia care experiences, illuminate areas for refinement and application, and support further testing.

